# Glucagon and heart in type 2 diabetes: new perspectives

**DOI:** 10.1186/s12933-016-0440-3

**Published:** 2016-08-27

**Authors:** Antonio Ceriello, Stefano Genovese, Edoardo Mannucci, Edoardo Gronda

**Affiliations:** 1 Institut d’Investigacions Biomèdiques August Pi i Sunyer (IDIBAPS) and Centro de Investigación Biomedica en Red de Diabetes y Enfermedades Metabólicas Asociadas (CIBERDEM), C/Rosselló, 149-153, 08036 Barcelona, Spain; 2 Department of Cardiovascular and Metabolic Diseases, IRCCS Multimedica, Sesto San Giovanni, MI Italy; 3 Diabetology, Careggi Hospital, University of Florence, Florence, Italy

**Keywords:** T2D, Glucagon, Heart failure

## Abstract

Increased levels of glucagon in type 2 diabetes are well known and, until now, have been considered deleterious. However, glucagon has an important role in the maintenance of both heart and kidney function. Moreover, in the past, glucagon has been therapeutically used for heart failure treatment. The new antidiabetic drugs, dipeptidyl peptidase-4 inhibitors and sodium-glucose co-transporter-2 inhibitors, are able to decrease and to increase glucagon levels, respectively, while contrasting data have been reported regarding the glucagon like peptide 1 receptors agonists. The cardiovascular outcome trials, requested by the FDA, raised some concerns about the possibility that the dipeptidyl peptidase-4 inhibitors can precipitate the heart failure, while, at least for empagliflozin, a positive effect has been shown in decreasing both cardiovascular death and heart failure. The recent LEADER Trial, showed a significant reduction of cardiovascular death with liraglutide, but a neutral effect on heart failure. A possible explanation of the results with the DPPIV inhibitors and empagliflozin might be related to their divergent effect on glucagon levels. Due to unclear effects of glucagon like peptide 1 receptor agonists on glucagon, the possible role of this hormone in the Leader trial remains unclear.

## Glucagon and glucose metabolism

Glucagon was identified as a pancreatic contaminant at the time of the discovery of insulin; it received the name of glucagon (GLUCose AGONist substance) because it was thought to be a glucose agonist [1]. This “long-known opponent” [2] of insulin in glucose homeostasis is a 29-amino acid hormone secreted by the α-cells of the pancreas. Its secretion is strictly correlated to the blood glucose levels: low levels of blood glucose in the fasting state determine secretion of glucagon and inhibit insulin secretion. Consequently, glucagon secretion restores glucose levels through hepatic glycogenolysis and gluconeogenesis, along with inhibition of glycogenesis.

Glucagon secretion is regulated by insulin and somatostatin, (as the main paracrine/endocrine inhibitors), glucose, glucagon-like peptide-1 (GLP-1), amylin, leptin, fatty acids, ketone bodies—all inhibiting glucagon secretion, glucose-dependent insulinotropic peptide (GIP), amino-acids (as l-arginine, leucine)—stimulating glucagon secretion, and by the autonomic nervous system. Meier et al. [3] demonstrated that also glucagon-like peptide-2 (GLP-2) stimulates glucagon secretion.

Some of the drugs often used in patients with type 2 diabetes (T2D), such as furosemide or acetylsalicylic acid, may influence prostaglandins (PG) synthesis, mainly PGE, which in turn control glucagon release [4]. Also, stressful stimuli, as hypovolemia, stimulate glucagon secretion [5].

It has been known for a long time that large meals containing only proteins increase whereas meals rich in carbohydrates decrease, glucagon secretion [6].

Insulin modulates glucagon secretion while physiological levels of glucagon stimulate insulin secretion [1, 2, 7].

Glucagon receptors are mainly located in the liver (hepatocytes and Kupfer cells) and kidney. They may be found also, at a lesser degree, in the endocrine pancreas (β- and α-cells), heart, adipocytes, gastrointestinal (GI) tract, brain, adrenal glands, lymphoblasts, retina and placenta [1, 2, 8]. Intense physical exercise, hypercorticism and stimulation of the ventro-medial hypothalamus determine an increase of glucagon secretion [6].

## Glucagon beyond glucose metabolism

Glucagon has been used not only as an inotropic agent and vasodilator but also as an inhibitor of the smooth muscle activity of the GI tract (including as an aid for radiological examinations) [9].

### Pleiotropic actions of glucagon

Today, besides its actions on glucose metabolism, glucagon is known to have other relevant effects [2, 3, 8]:

- On lipid metabolism: decreased plasma cholesterol, total esterified fatty acids, decreased hepatic synthesis of triglycerides and apolipoproteins. In addition, glucagon determines lipolysis in white adipose tissue.
- Increased ketone-body production and fatty acid oxidation.
- Increased energy expenditure and thermogenesis by an increased oxygen consumption, blood flow and heat production in brown adipose tissue.
- Decreased food intake, with reduction of meal size, increased satiation (possibly mediated through ghrelin) and decreased gastric emptying.
- Regulation of secretion of other hormones, such as insulin, ghrelin, somatostatin, cortisol and growth hormone.
- Retinal function: loss of retinal function and of visual acuity and retina cells death have been described after the disruption of glucagon receptor (gcgr) gene and were directly correlated with the degree of hypoglycaemia in rodent models.

The above-described effects make glucagon an attractive therapeutic target in obesity, eating and lipid disorders [3].

### Glucagon and the heart

Along with the key actions of glucagon in glucose homeostasis, since the 60s, (after Unger adapted the radioimmunoassay to measure glucagon), human and animal experiments have highlighted direct actions on the heart of physiological levels of glucagon [3, 7].

In humans it has been proved that acute glucagon administration exerts a positive action on cardiovascular performance either increasing cardiac index, either decreasing peripheral vascular resistances [10]. Moreover, the positive enhancement of cardiovascular performance is comparable to what has been observed in cat and dog, with persistence of action despite beta-receptor blockade with propranolol [11].

In the non-failing heart, glucagon determines a rise in heart rate, almost without changes in cardiac output and auricular pressure; in the failing heart, it increases heart rate and cardiac output, together with a dose-dependent increase in coronary blood flow and oxygen consumption [12]. These actions of glucagon are mediated through cyclic AMP (cAMP).

Several factors affect glucagon actions on the heart. Among them, as seen above, the most important for our topic is heart failure (HF). In this context, the type of HF, its severity and chronicity are relevant [12, 13]. In fact, a greater severity of HF is associated with a smaller hemodynamic response to glucagon. In addition, glucagon determines a weaker response in chronic than in acute HF [12, 13].

The inotropic effects of glucagon are more potent in the ventricle than in the atrium, [7]. It does not increase irritability of the myocardium and it is active in the presence of digitalis and propranolol. Thus, attempts for a therapeutic use of glucagon have been made in resistant cardiac failure, myocardial infarction, hypotension following cardiac operations, intoxication with β-/calcium channel blockers and heart block [7, 14–16].

Glucagon also facilitates the atrio-ventricular conduction and its inotropic action is accompanied by an anti-arrhythmogenic effect (which, might be due partially to an increased insulin-mediated uptake of potassium (K^+^) by the myocardium following glucagon administration) [12, 14]. Glucagon has an important effect on sino atrial node rate [17] and the antiarrhythmogenic effect of glucagon has been several times reviewed [18–23].

Furthermore, glucagon is able to decrease histamine-induced cardiac injury during reperfusion [2] and restores the pressure of the coronary perfusion during ischemic vasodilation [24].

As an inotropic agent, glucagon increases the work of the heart and, consequently, it increases oxygen consumption, lipolysis and beta-oxidation of lipids [1]. It is noteworthy that both insulin and glucagon increase fuel availability in the heart. In animal studies, glucagon, similarly to insulin, increases glycolysis and glucose oxidation through phosphatidylinositol 3-kinase-dependent and adenylate cyclase- and cAMP-independent pathways [7].

As hyperglucagonemia determines an increased availability of substrate and improves cardiovascular (CV) performance (crucial in a physiological stress response), this hormone is considered today to be a stress hormone [5].

### Glucagon and the kidney

In the kidneys, glucagon at relatively high doses, induces vasodilation with a concomitant increase in renal plasma flow (RPF), glomerular filtration rate (GFR) and electrolyte excretion. These changes are more evident in patients with diabetes, possibly due to the modified insulin:glucagon ratio; the administration of insulin, which normalizes this ratio, brings GFR and RPF close to normal values.

In respect to the electrolytes, glucagon is responsible of the increase of natriuresis in the fasting state. During starvation glucagon is increased and insulin is decreased; re-feeding with carbohydrates has an anti-natriuretic effect [12].

Glucagon determines initially a transient increase in plasma K^+^ levels (partially due to the hepatic glycogenolysis), followed by hypopotassemia determined by an increased uptake (muscles, liver) induced by insulin.

Glucagon also increases the urinary excretion of calcium, phosphate and zinc [12].

The direct action of glucagon (and vasopressin) in protein-induced hyperfiltration is well established now [25]. Glucagon plays an important role in the excretion of nitrogen end products (increased urea synthesis in the liver and urea excretion in the kidney), while vasopressin concentrates these products in a hyperosmotic urine and producing water economy. In the absence of any of these two hormones, glomerular hyperfiltration is not possible [25]. In conclusion, glucagon is a relevant component of the close relationship between heart and kidney function, aiming to maintain the continuum pressure volume circulatory balance.

## Glucagon in type 2 diabetes

Schematically, type 2 diabetes (T2D) is characterized by: β-cell failure, α-cells insulin resistance and decreased incretin effect.

- *β-cell failure* due to partial loss of β-cell mass and β-cell dysfunction, influenced by a genetic background and by chronic exposure to gluco- and lipotoxicity, amylin and advanced glycation end-products (AGEs).
- *α-cells insulin resistance* the so called by Unger and Orci, “paracrinopathy” and T2D “a bi-hormonal disorder”. In T2D, α-cells might be resistant to the inhibitory effect of insulin [26] or to other β-cell secretory products such as zinc or γ-aminobutyric acid [7].

Consequently, T2D is characterized by fasting hyperglucagonemia and impaired glucose-induced glucagon suppression in the post-prandial state (insulin/glucagon concentration inversely related in the post-prandial state). Mainly due to β-cell apoptosis, β**/**α-cell ratio is altered, contributing to a decreased insulin/glucagon ratio. Also, β-cell may de-differentiate to progenitor pluripotent cells that may release glucagon and somatostatin, thus further decreasing insulin/glucagon ratio [3, 7]. In this context, it is clear that glucagon is a key hormone worsening the metabolic consequences of insulin deficiency [27]. Type 2 diabetes is also characterized by a decreased incretin effect: in T2D, glucose-dependent insulinotropic peptide (GIP) and GLP-1 account only for <20 % of the postprandial insulin response. While GLP-1 action is relatively preserved, β-cell impairment determines a decrease in the insulinotropic action of GIP; the following hyperglycaemia further downregulates the GIP receptor in β-cells, aggravating the impairment of the incretin effect, creating a vicious cycle [1].

A decrease in incretin action may affect the crosstalk between AGEs-RAGE axis, favouring the appearance of complications [28]. It is worthy of interest that when hepatic glycogen content is low, glucagon response to insulin-induced hypoglycemia is reduced [29]. In this view, glucagon has been recently suggested as the key feature of type 2 diabetes [30].

## New antidiabetes treatments and glucagon

The increased glucagon/insulin ratio is a key factor in the pathogenesis of hyperglycaemia in T2D; the lack of glucagon suppression in the post-prandial state leads to post-prandial hyperglycaemia, whereas inappropriate levels of glucagon in the fasting state leads to increased hepatic glucose production and fasting hyperglycaemia.

Consequently, addressing glucagon seemed an attractive treatment for T2D by either suppression of glucagon secretion or by blocking gcgr. Monoclonal glucagon antibodies, glucagon receptor antagonists (peptide and non-peptide) and molecules targeting the expression of gcgr have all been tested as potential treatments for T2D [7].

However, research on those agents encountered a number of major obstacles, most notably a limited efficacy, the risk of iatrogenic hypoglycaemia, and other safety issues related to lack of specificity of glucagon blockade, immunogenity and toxicity [31].

Interestingly, the reduction of glucagon levels should be one of the main mechanisms of action of some of the recent antidiabetes drugs (ADD) for T2D, such as GLP-1 receptor agonists (GLP-1 RA) and inhibitors of dipeptidyl peptidase-4 (DPP-4i) [31].

*GLP-1RA* increase insulin synthesis and secretion in a glucose-dependent manner and decrease glucagon levels probably; this latter effect could be exerted through somatostatin or neural regulation, as the α-cell do not show GLP-1 receptors [1, 31].

Additionally, GLP1-RA slow gastric empting, decrease appetite, induce satiety and potentially, inhibit β-cell apoptosis [32]. They have favorable effects on body weight and body composition (decreasing mainly the visceral fat) and plasma lipids. In animal models, long-term treatment with GLP1-RA also increase β-cell mass [33, 34].

However, it has been shown that glucagon and GLP-1 co-agonism has a significant greater efficacy than GLP-1 RA monotherapy on body weight, body composition, glucose and lipid metabolism, including the reversal of hepatic steatosis, making this combination therapy an attractive and promising treatment for obesity and metabolic syndrome [2].

Short-acting GLP-1 RA (exenatide and lixisenatide) lower mainly post-prandial glucose, partly by inhibiting gastric empting: conversely longer-acting molecules of the class (albiglutide, dulaglutide, exenatide LAR, liraglutide) target predominantly fasting plasma glucose through their insulinotropic and glucagonostatic actions [33, 34]. The durability of the glucagonostatic effect in the long-term treatment with GLP-1 RA has been investigated for liraglutide in the LIBRA trial. Quite surprisingly, chronic treatment with liraglutide in patients with early T2D has been associated with an increased post-challenge glucagonemia [35]. The real effects of GLP-1RA on glucagon probably remains to be further elucidated, considering that also Albiglutide does not affect glucagon secretion during hypoglycemia [36].

*DPP-4i* increase the duration of action of the endogenous GLP-1 through the inhibition of DPP-4 enzyme. Consequently, the GLP-1 levels are increased although the stimulation of the GLP-1 axis is not so potent as that determined by GLP1-RA. This action is sufficient to determine a moderate reduction of blood glucose, with no effects on body weight, no risk of severe hypoglycemia, and no gastrointestinal side effects. DPP-4i also induce an increase in circulating levels of GIP, which is another one of the substrates of DPP-4. The reduction in glucagon levels observed during treatment with DPP-4i results from the increase of both GLP-1 and GIP [34, 37].

Worth to mention, mainly in the context of this article, is that the incretins have beneficial effects on the CV system by decreasing blood pressure, improving left ventricular function and endothelium-dependent vasodilation and by increasing the endothelial progenitor cells [38].

*SGLT-2i* are the last approved class of ADD, which inhibit glucose reabsorption in the proximal renal tubule, independently of insulin secretion or action. Consequently, glucose is lost in the urine and glycaemia is decreased [34].

Regarding glucagon secretion, in opposition with GLP-1 RA and DPP-4i, SGLT-2i increase plasma glucagon.

In the kidney, glucagon controls the rate of filtration, urea excretion and the reabsorption of water through direct and indirect mechanisms, even though its role in renal glucose output is unclear. In an animal model, long-term infusion of glucagon induced hypertension, hypertrophy, and increased proliferation of mesangial cells [7].

A non-selective inhibitor of glucose reabsorption in the proximal nephrons which targets both SGLT-1 and SGLT-2, phlorizin, was increasing glucagon levels, an effect confirmed by SGLT-2i [3]. The association between SGLT-2 inhibition, increased endogenous glucose production (EGP) and glucagon during euglycaemia has not been expected. It has been demonstrated that the SGLT-2 transporters are expressed not only in the proximal tubules but also in the α-cells, but not in β-cells. The inhibition of SGLT-2 directly stimulates glucagon secretion through the activation of K_ATP_ channel [39, 40].

Since the mechanism of action of SGLT-2i is independent of β-cells and different from that of the other existing classes of ADD, it has been suggested that SGLT-2i might be combined with other drug(s) and mainly with DPP4i; this combination therapy is well-tolerated and not accompanied by the most common seen side-effects: hypoglycaemia or weight gain. Also, DPP4i increase insulin secretion and decrease glucagon; so, potentially they block the compensatory EGP seen with SGLT-2i and increase their glucose-lowering capacity [38, 41, 42].

## Cardiovascular outcome trials and glucagon

After the CV safety concerns raised by some ADD either in development or marketed (e.g. muraglitazar, rosiglitazone) and by the results of some clinical trials (e.g. ACCORD), the US Food and Drug Administration (FDA) issued in 2008 the “FDA Guidance for Industry” focusing on the CV risk assessment of ADD. Consequently, post-approval, there is the requirement to perform a CV outcome trial (CVOT) [43, 44], which has as primary endpoint major adverse cardiac events (MACE) such as: CV death, non-fatal MI, and non-fatal stroke—the “canonical” CVD endpoints; some CV outcome trials include also heart failure, unstable angina requiring hospitalization, amputation, and revascularization procedures—the “hard” CVD endpoints [45].

It should be mentioned that the actual “FDA Guidance for Industry”, issued in 2008, is not requiring hospitalization for HF as a primary/secondary outcome parameter.

In Saxagliptin Assessment of Vascular Outcomes Recorded in Patients with Diabetes Mellitus (SAVOR)—Thrombolysis in Myocardial Infarction (TIMI) 53 (SAVOR-TIMI 53) trial the major secondary endpoint was a composite of CV death, myocardial infarction (MI), stroke, hospitalization for unstable angina, coronary revascularization or HF. A total unexpected finding was the increased hospitalization for HF in T2D subjects on saxagliptin versus placebo (HR 1.27, 95 % CI 1.07–1.51, *p* = 0.007) [46].

In the primary publication of the Examination of Cardiovascular Outcomes with Alogliptin versus Standard of Care (EXAMINE) trial, despite the fact that 28 % of T2D subjects had congestive HF at baseline, no information on HF outcomes has been reported [47, 48]. Unfortunately, hospitalization for HF was not planned as a stand-alone endpoint in the EXAMINE, so that a specific analysis on this outcome could not be reported in the primary publication. (Hospitalization for heart failure 106 out of 2701 events in the alogliptin arm compared to 89 out of 2679 events in the placebo arm: HR 1.19, 95 % CI 0.89–1.58). In a post hoc analysis [49], there was a trend (*p* = NS) for increased hospitalization for HF with alogliptin versus placebo. The fact that the trend toward increase of hospitalizations for HF in the overall cohort was not statistically significant is not surprising, considering the relatively small sample size of subjects in this trial. However, in the same study, an increased hospitalization for HF has been reported in patients without a previous history of HF treated with alogliptin, but not in patients with a previous history of HF [49]. Standl and Schnell show the limitation of this post hoc analysis (different composite endpoints) and point out the similarities between SAVOR TIMI 53 and EXAMINE (in both studies, in patients without history of HF and treated with DPP4i the rate of HF was increased) [50]. In addition, vildagliptin in ventricular dysfunction diabetes (VIVIDD) trial, showed an unanticipated rise in left ventricular end-diastolic volume, end-systolic volume, and cardiac stroke volume [50]. On the other hand, while an increase of HF has been also reported with sitagliptin use [51], no increase in the incidence of hospitalization for HF was observed with sitagliptin in the trial evaluating cardiovascular outcomes with sitagliptin (TECOS) trial [52, 53], or with incretin therapy in a recent observational study with a large cohort of T2D patients [54]. Noteworthy, the T2D population included in each of these outcome trials was different: if in TECOS, these subjects were with an established CVD, in SAVOR TIMI they had a history or were at risk for CVD and lastly, in EXAMINE they had an acute coronary syndrome within the previous 15–90 days [46, 47, 52]. This fact might (partly) explain the different results on the hospitalization for HF. Anyhow, recently the AHA stated that this could be a class effect [55].

Surprisingly, the EMPA-REG study with empagliflozin, a SGLT-2i, showed a CV protective effect of this compound [56]. Apart the osmotic diuresis, there are proposed many other mechanisms behind CV protection of SGLT2i: improved insulin sensitivity, decreased blood pressure and arterial stiffness, body weight and visceral adiposity, decreased inflammation and oxidative stress [40].

Both results of CVOT on hospitalization for HF in T2D unfavorable with saxagliptin in SAVOR-TIMI 53 and favorable ones with empagliflozin in EMPA-REG might be explained, at least in part, by their impact on glucagon levels: DPP-4i reduce, and SGLT-2i increase, glucagon levels [57].

The reduced risk of hospitalization for heart failure with empagliflozin [56], which is present in patients with and without baseline heart failure [58], might be partly explained by a direct enhancement of myocardial function, determined by the increased levels of glucagon, and by its natriuretic effect. In addition, the beneficial effect of glucagon on disturbances of cardiac rhythm could be partly responsible for the reduction of CV mortality with empagliflozin. Conversely, the reduction of glucagon levels during treatment with DPP4i could precipitate HF in individuals with unstable hemodynamic compensation. Indeed, in SAVOR-TIMI 53 the increased risk for hospitalization for HF with saxagliptin was observed in patients with the highest N-terminal pro B-type natriuretic peptide quartile and in those with chronic kidney disease [59], a trend confirmed also in the EXAMINE [49], while no data on N-terminal pro B-type natriuretic peptide are available for the TECOS.

Furthermore, the protective effects on diabetic kidney disease of empagliflozin [60] could also be partly explained by the increase of glucagon, considering its effects on the kidney described before.

It has been shown that GLP-1 RA have an insulinotropic and glucagonostatic effect [32, 34]. However, the data regarding GLP-1RA and glucagon need to be more clarified: recently, in the LIBRA trial it has been demonstrated that long-term treatment with liraglutide in early T2D has been associated with an increased post-challenge glucagonemia [35], while for Albiglutide the evidence is that it does not affect glucagon secretion during hypoglycemia [36]. Liraglutide reduced the cardiovascular mortality in the LEADER trial, but with a neutral effect on HF [61]. Both liraglutide and albiglutide were also not effective in improving HF in two recent trials in type 2 diabetes with advanced HF [62, 63]. At the state of the art, in our opinion, a possible involvement of glucagon in the results on the mortality of the LEADER trial cannot be either claimed or excluded.

## Conclusions

The glucagon inotropic action in human heart is well represented in not failing heart, but progressively declines in the failing heart, becoming undetectable in severe heart failure condition [13]. Therefore, the plasma levels of glucagon may contribute to maintain the heart function when the HF is not severe, which is the case of people recruited in the studies we mentioned. This means that glucagon might be very important for the heart and cardiovascular system in type 2 diabetes. This is a paradox, considering that this hormone has been always considered harmful in diabetes. Future studies are needed to endorse or not the role of glucagon on heart, and may be also on kidney, in T2D.

## Abbreviations

AGEs: advanced glycation end-products
ADD: antidiabetes drugs
BNP: brain natriuretic peptides
CV: cardiovascular
CVD: cardiovascular disease
CVOT: CV outcome trial
DPP-4: dipeptidyl peptidase-4
DPP-4i: inhibitors of dipeptidyl peptidase-4
EGP: endogenous glucose production
GI: gastrointestinal
GFR: glomerular filtration rate
GLP-1: glucagon-like peptide-1
GLP-1 RA: GLP-1 receptor agonists
GLP-2: glucagon-like peptide-2
Gcgr: glucagon receptor
GIP: glucose-dependent insulinotropic peptide
HF: heart failure
MACE: major adverse cardiac events
MI: myocardial infarction
PG: prostaglandins
RPF: renal plasma flow
SGLT-2i: sodium-glucose co-transporter-2 inhibitors
T2D: type 2 diabetes
